# Histological Comparison of the Human Trunk Skin Creases: The Role of the Elastic Fiber Component

**Published:** 2016-03-29

**Authors:** Andreas Mallouris, Despoina Kakagia, Andreas Yiacoumettis, Thivi Vasilakaki, Aggeliki Drougou, Maria Lambropoulou, Constantinos Simopoulos, Alexandra K. Tsaroucha

**Affiliations:** ^a^Aretaeion Hospital, Nicosia, Cyprus; ^b^Second Surgery Department, Democritus University of Thrace, Alexandroupoli, Greece; ^c^First Surgery Department, Democritus University of Thrace, Alexandroupoli, Greece; ^d^Plastic Surgery Department, Iaso General Hospital, Athens, Greece; ^e^Pathology Department, Tzanio General Hospital, Piraeus, Greece; ^f^Pathology Department, Iaso General Hospital, Athens, Greece; ^g^Pathology Department, Democritus University of Thrace, Alexandroupoli, Greece

**Keywords:** skin crease, skin fold, inframammary, infragluteal crease, elastic fibers

## Abstract

**Objective:** Skin creases are features of major anatomical, morphological, surgical, and functional interest. This study focuses on the histological comparison of creases of the trunk and participation of the elastic fibers in their formation. The histological structure is a key consideration for the reconstructive planning of the relevant area and its knowledge may contribute in such direction. **Methods:** Fresh cadaver specimens were collected from the inframammary (*n* = 15), infragluteal (*n* = 16), and inguinal creases (*n* = 14), the anterior axillary fold (*n* = 14), and the surrounding skin (*n* = 10). Specimens were fixed in 10% buffered formaldehyde. Collagen and muscle fibers were stained by Masson Trichrome and Van Gieson stains, reticular and collagen type III fibers by Reticulin stain, and elastic fibers by Verhoef and Orcein stains. **Results:** Skin creases of the trunk present well-defined dense bundles of collagen fibers, creating a beehive pattern with broad attachment to the dermis and denser in deeper sites related to the fascia of the underlying muscle. The elastic fibers participate in the collagen pattern and radiate in a parallel pattern in the reticular dermis and in a perpendicular fashion in the papillary dermis. The skin surrounding the creases lacks such organization. **Conclusions:** Creases of the trunk are formed by well-organized collagen bundles in a beehive pattern, attached to the dermis and related to the underlying muscle fascia. The elastic fibers participate in this structure and radiate in a parallel fashion in the reticular dermis and perpendicularly in the papillary dermis.

Skin creases of the human body^1^ are features of great anatomical, developmental, functional, and surgical interest. Skin creases are highly variable regarding their histological and structural characteristics, and this is primarily determined by their location and anatomical site. Their histological structure is a key consideration during surgical planning in skin and soft-tissue reconstructive surgery.^2^^,^^3^

As described by Lockwood,^4^ connective tissue of the dermis along with the superficial fascia system (SFS) is responsible for the characteristic pattern of lines^5^ of the skin, as well as for the fixed and permanent nature of skin creases of the trunk (Fig 1).

There is paucity in the literature regarding the role of the elastic fibers component in skin crease formation.^2^^,^^6^^,^^7^ Several syndromes associated with disorders of the connective tissue involving the elastic fibers present with structural abnormalities concerning the skin creases.^8^^,^^9^

The aim of this study was to investigate and compare the histological structure of human trunk skin creases, especially in terms of the role of the underlying dermal elastic fibers. Improving our understanding on this area may be of paramount importance in surgery.

## MATERIALS AND METHODS

For the purpose of this study, 69 specimens from fresh cadavers were collected. The study complied with guidelines on experimental research set by the University Bioethics Committee and the Declaration of Helsinki, and it commenced following the approval of the Bioethics Committee.

According to the inclusion criteria regarding the selection of cadavers, only whites without a history of surgery or trauma at the relevant area, nor any disorder of the connective tissue, were included. This study investigated skin creases of the trunk.

Dissection was standardized and performed using no. 10 and 15 blades. The collected specimens were skin sections that included the relevant skin crease centrally (along the longitudinal axis) and approximately up to 2 cm of the additional tissue, superior and inferior to the relevant crease. The full-thickness specimens included all layers of the skin, subcutaneous fat with fascia, the fascia plane of the underlying muscle, and part of the muscle tissue, as well as the periosteum of the underlying osseous substrate when anatomically related to the skin crease. The lateral and medial borders of the specimens were tagged so that their original orientation on the human body remains identifiable. The specimens were then fixed in 10% buffered formaldehyde.

Fifteen specimens of the inframammary crease were collected, from 11 female and 4 male cadavers, aged 45 to 82 years. The deep margin of dissection was over the periosteum and the intercostal plane, medially extended to the sternum and laterally to the anterior axillary line (Fig 2*a*).

The infragluteal crease was studied in 16 fresh cadaver specimens, 12 female and 4 male cadavers, aged 42 to 80 years. Tissue samples were dissected to the fascia plane of the underlying muscle and were oriented in relation to their medial and lateral borders.

Fourteen specimens were collected for the study of the anterior axillary fold, 8 female and 6 male cadavers, aged 45 to 76 years. Tissue samples were dissected up to the suprafascial plane.

Tissue samples were obtained from 14 fresh cadavers, 7 female and 7 male cadavers, aged 42 to 76 years, for the study of the inguinal crease, and they were also dissected down to the suprafascial plane.

Ten specimens were also collected, from approximately 2 cm superior and 2 cm inferior to the central site of the relevant skin crease (Fig 2*a*), 5 superior or inferior to the inframammary crease, and 5 to the infragluteal crease.

The specimen study was conducted by staining with hematoxylin and eosin as a standard staining, using Masson Trichrome stain for collagen and muscle fibers, Van Gieson stain for collagen and muscle fibers, and Reticulin stain for reticular and collagen type III fibers. For the study of the elastic fibers, Verhoef and Orcein stains were applied.

Further evaluation of the specimens included the observation of the structure of the tissue with a Nikon Eclipse 50i microscope. The type of the digital camera used for the documentation of microscopy findings was Nikon Sight ds-L1.

## RESULTS

Microscopic evaluation of skin specimens revealed normal dermis and appendages in all cases. In all sections of the inframammary crease specimens, no breast parenchyma was present. In the subcutaneous tissue, there was a well-defined network of dense collagen fibers that create a beehive pattern of the subcutaneous fat (Fig 3). This pattern is denser in the deeper sites in relation to the fascia of the underlying muscle (Figs 4, 5*a*, and 5*b*). The collagen fibers network has a broad base of attachment to the dermis. In the sections from the medial sites of the crease, the beehive pattern has the same structure as that described earlier but appears looser. However, it becomes denser in the deeper sites. In the sections of the lateral sites of the crease, the beehive pattern is equally well organized, with broad base attachments of the collagen bundles to the dermis (Fig 6). In some of the cases, the bundles are thinner than those at the other sites. The elastic fibers participate in the formation of the collagen pattern and radiate in a relatively parallel pattern in the reticular dermis and in a perpendicular fashion in the papillary dermis (Fig 7).

In the subcutaneous tissue of the infragluteal crease, there is a well-defined network of dense collagen fibers that create a beehive pattern. The collagen bundles are thick and dense, well-organized, and characterized by the presence of a significant number of fibroblasts, particularly at the sites reflecting the center of the crease (Fig 8). In sections of the specimens from the medial and lateral sites of the crease, the collagen pattern is thinner, the presence of fibroblasts is not as significant as in the central sites of the crease, and the participation of the reticular fibers is less. The elastic fibers are thick in the collagen pattern, denser in sections that reflect from the center of the crease. They radiate into the dermis in a parallel pattern in the reticular dermis and in a perpendicular fashion in the papillary dermis.

The findings from the anterior axillary fold specimens show a less organized pattern structure of fibrocollagenous bundles compared with the earlier described structure of the inframammary and infragluteal creases. The bundles are fragmented and not in a parallel pattern. There is absence of any organized structure of the elastic fibers in the dermis along the anterior axillary fold (Fig 9).

In the specimens of the inguinal crease, there is a beehive pattern of fibrocollagen network that is thinner than the structure of the inframammary and infragluteal creases. The bundles are organized in a looser pattern (Fig 10). The elastic fibers are long and run along the collagen bundles in a parallel course into the reticular dermis (Fig 11).

The skin specimens superior and inferior to the skin creases assert that collagen and elastic fibers have a rather random pattern compared with the well-organized network of the fibroelastic bundles in the skin of the creases sites. The elastic fibers are fewer and have a parallel pattern in the dermis (Fig 12*a*) in the skin inferior and superior to the infragluteal crease and are fewer in number and placed in a parallel pattern in the dermis (Fig 12*b*) in the skin superior and inferior to the inframammary crease.

## DISCUSSION

Skin crease is a visible, fixed, and permanent anatomical structure,^10^ with attachment to the underlying structures.

The creases are important structures and define the contour of the relevant area. Their significance reflects on the morphology and anatomy, and they define the surgical approach of the relevant area by providing fixed regional landmarks that have to be respected during reconstruction intraoperatively.

In addition, another important objective of this study is to understand and compare the specific structural characteristics of creases and folds in order to reflect on their functional role in overlying the motile parts of the musculoskeletal system (eg, major joints or muscle groups) as well as on their special configuration and its role in the contour and appearance of the body. Consequently, structural understanding leads to functional and formative insights, which, in turn, are of paramount importance during reconstruction and surgical treatments.

In the literature, often the terms “crease” and “fold” refer to the same anatomical structure.^11^ Skin crease is a permanent line, whereas skin fold is the redundancy of the skin over this permanent line. The accurate terminology with regard to these anatomical structures is important and is relevant to their anatomical and histological characteristics. In addition, a fold is generally more complex structurally and is defined by all 3 dimensions of the adjacent skin and soft tissue almost equally, obtaining certain characteristics due to the physical properties of the surrounding tissues, for example, the natural hanging of tissue due to gravity. Conversely, a crease defines adjacent regions primarily on the 2-dimension plain or at least not to such an extent in the 3 dimensions that could alter the contour of the body.

The surface of the body is characterized by a lattice pattern of lines^5^ that increase the body surface, help achieve uniform distribution of the stress, as well as enhance the ability of the skin to stretch and recoil.^5^ Depending on the body area and its functional requirements, the pattern of lines varies. The skin creases are fixed and permanent lines,^10^ and they should not be confused with the lines of limited tension, nor the Langer lines.^5^

The connective tissue and the SFS, described by Lockwood,^4^ play a major role in the formation of skin creases of the trunk. The histology of skin creases in the face is different from the crease of the trunk, where the insertions of fibers from the mimic muscles into the dermis of the skin at the site of the crease play the most important role in their formation. The study by Patel et al,^2^ referring to the alar-facial crease,^1^ describes greater participation of the elastic fibers in the formation of the crease compared with collagen and muscle fibers. Barton and Gyimesi,^7^ in their study of the nasolabial crease,^1^ have observed dermal insertions of fibers from the mimic muscles of the upper lip along the skin of the nasolabial crease. The anatomist Jones^10^ has studied the skin creases of the hand and refers to the fact that they do not correspond to the underlying relevant joints. This is an observation also supported in the studies by Bugbee and Botte.^12^ These skin creases are characterized by strong attachments of the dermis of the crease to the underlying fascial system. Such knowledge not just contributes to the overall surgical planning from the approach and initial incision choices to the methodology of reconstructing both form and function (eg, by incorporating the correct muscle group fibers in a motile part of the face during facial reanimation).

The majority of available histological studies referring to the skin creases do not emphasize on the elastic fiber component with limited exceptions.^2^^,^^6^^,^^7^

Considering the fact that many syndromes with disorders of the connective tissue, including the elastic fibers, have clinical signs that affect the normal appearance of the skin creases,^8^^,^^9^ this study includes the elastic fibers component as an important part of the fibroelastic system that supports the formation and maintenance of skin creases.

**Figure 1:**
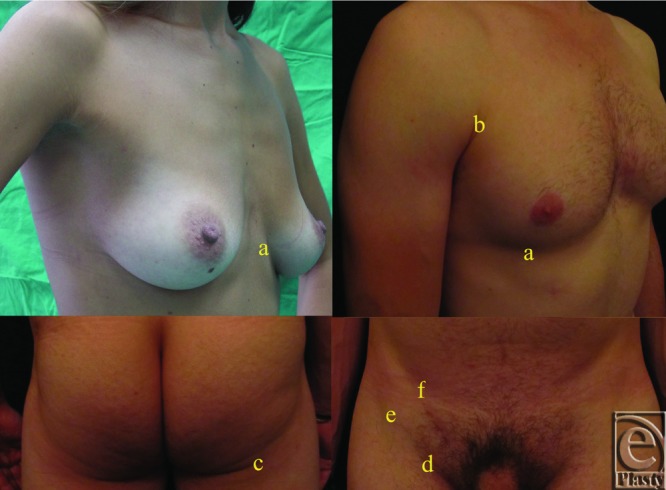
Skin creases of the trunk: (a) inframammary crease; (b) anterior axillary fold; (c) infragluteal crease; (d) inguinal crease; (e) oblique crease of the groin; and (f) suprapubic crease.

**Figure 2:**
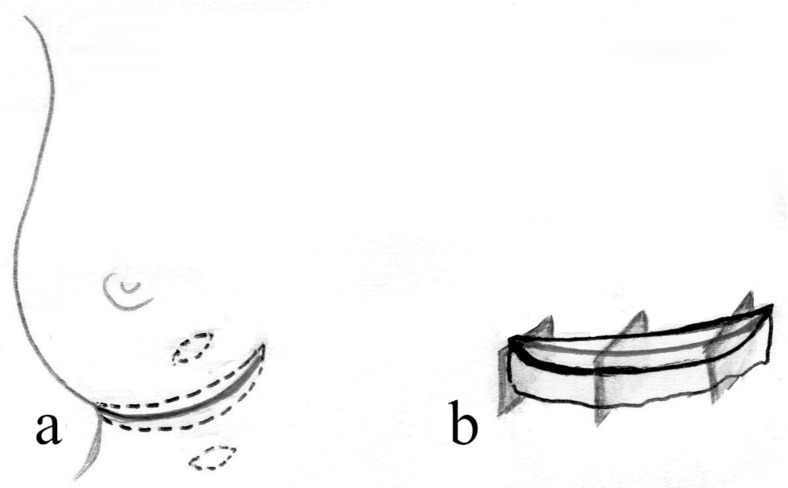
Dissection and orientation of the specimens. (a) The skin crease specimen extends from the medial to the lateral edge of crease. Specimens of the skin at a distance 2 cm superior and 2 cm inferior of the crease were also collected. (b) The specimens were examined with vertical dissections of the medial, lateral, and central sites of the crease.

**Figure 3:**
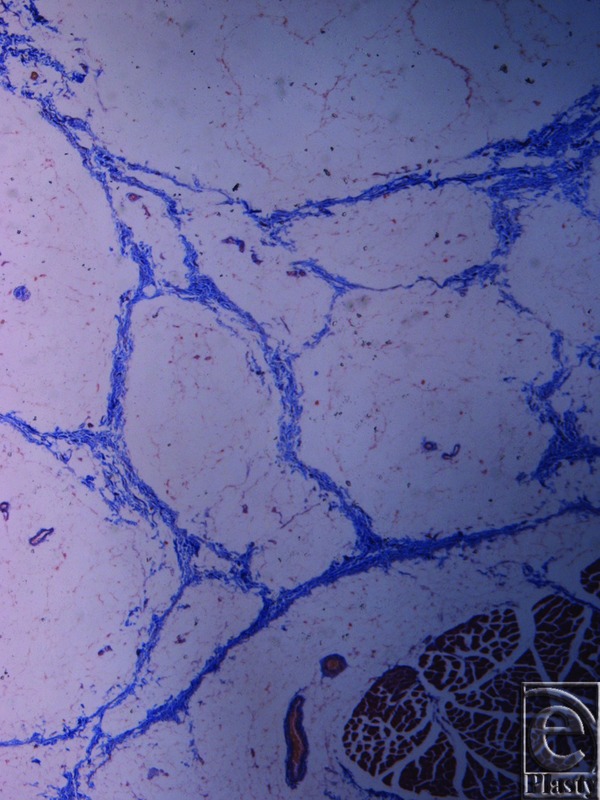
Inframammary crease. Masson trichrome stain ×25. The subdermal fat and the fibrocollagen bundles, stained in blue, are arranged in a beehive pattern. The collagen bundles originate from the muscle fascia.

**Figure 4:**
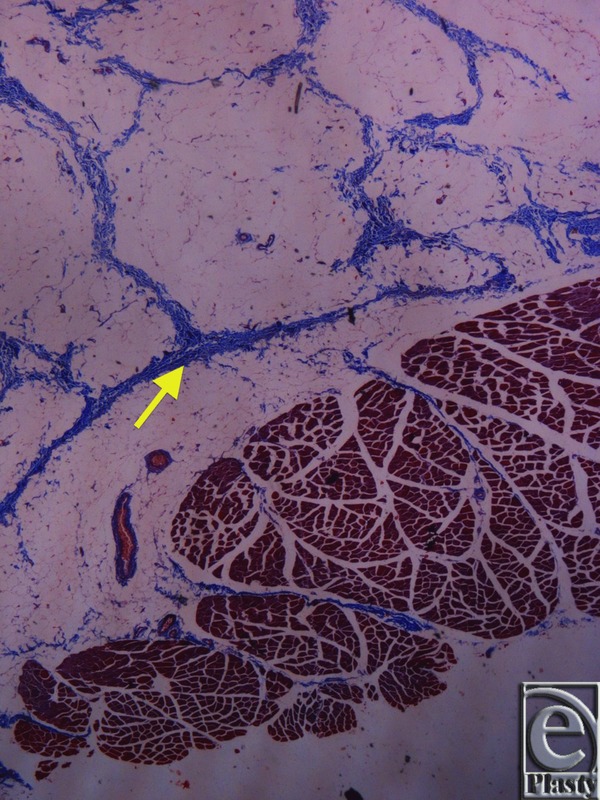
Inframammary crease. Masson trichrome stain ×25. The collagen fibers originate from the muscle fascia and continue their course in the fibrocollagen bundles in a beehive pattern.

**Figure 5:**
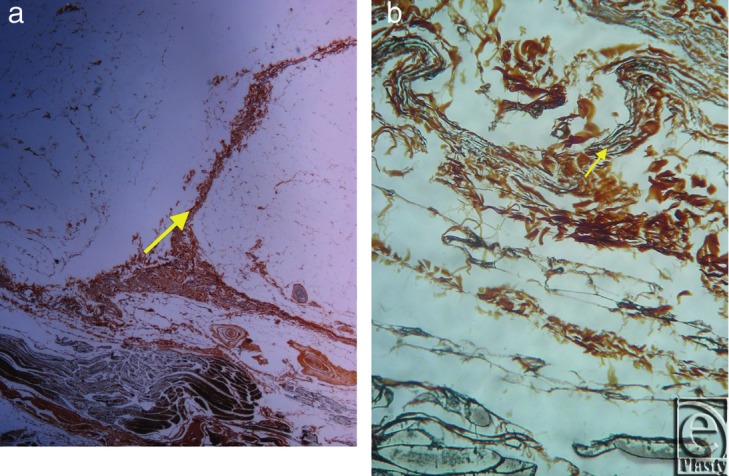
Inframammary crease. (a) Reticulin stain ×25. (b) Reticulin stain ×200. The collagen type III fibers are stained in black and radiate from the underlying muscle fascia.

**Figure 6:**
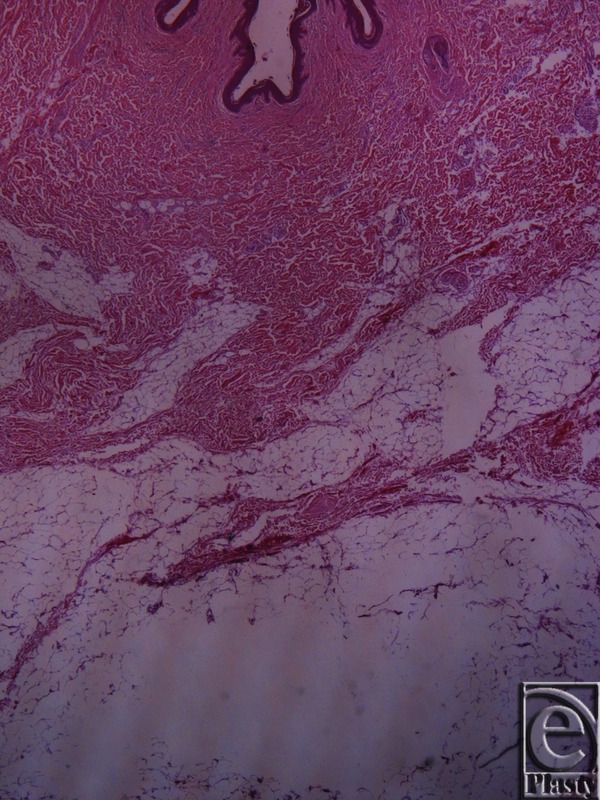
Inframammary crease. Hematoxylin and eosin ×25. The fibrocollagen bundles of the subdermal tissue are attached to the dermis with a broad base.

**Figure 7:**
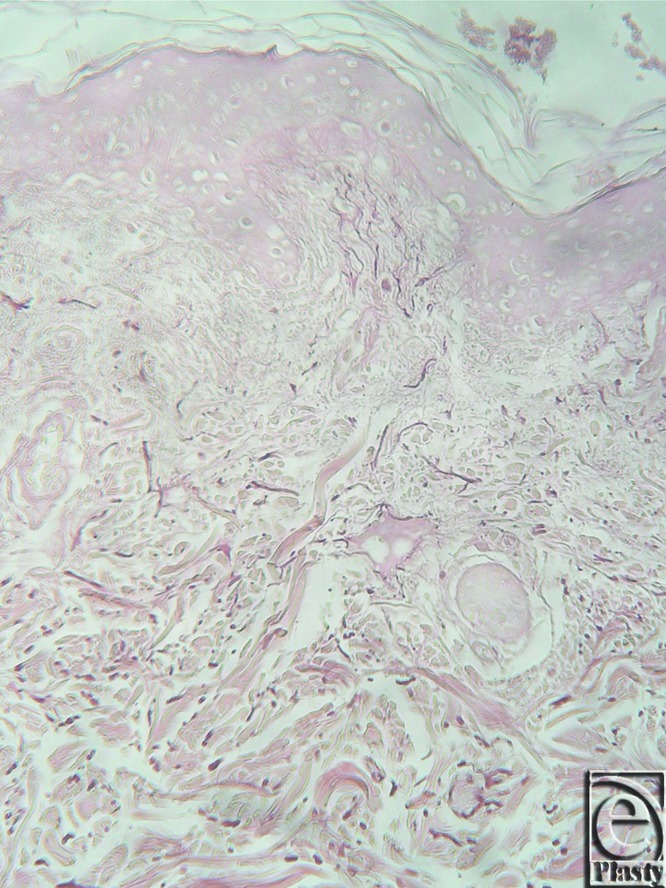
Inframammary crease. Orsein stain ×25. The elastic fibers have a parallel pattern in the reticular dermis and a perpendicular pattern in the papillary dermis.

**Figure 8:**
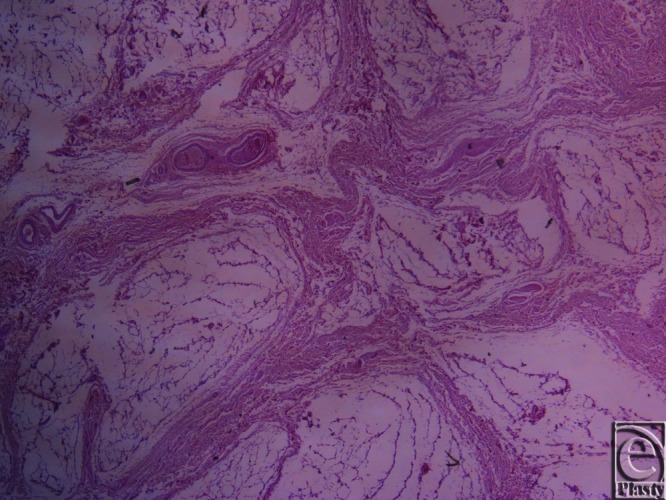
Infragluteal crease. Hematoxylin and eosin stain ×100. The subcutaneous fat and the dense and thick fibrocollagen bundles are arranged in a beehive pattern.

**Figure 9:**
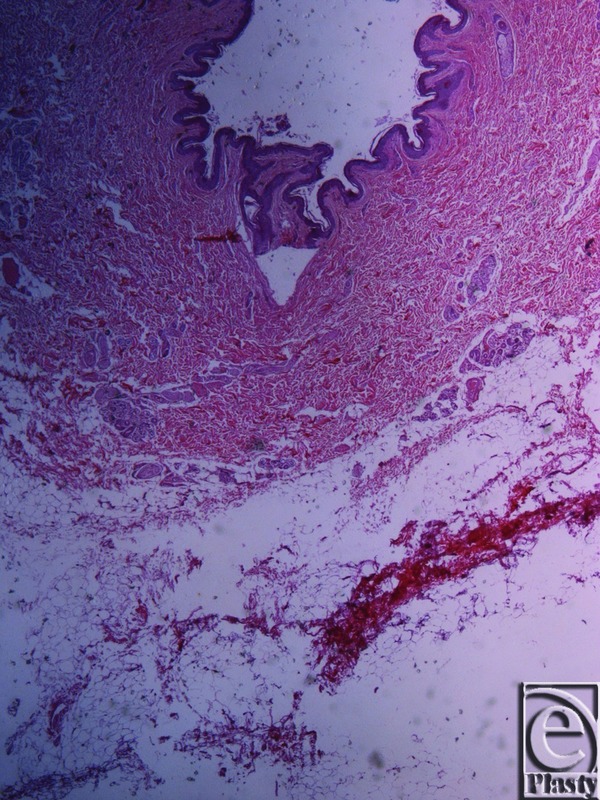
Infragluteal crease. Hematoxylin and eosin stain ×25. The fibrocollagen bundles are less organized without broad attachments of the fibers to the dermis.

**Figure 10:**
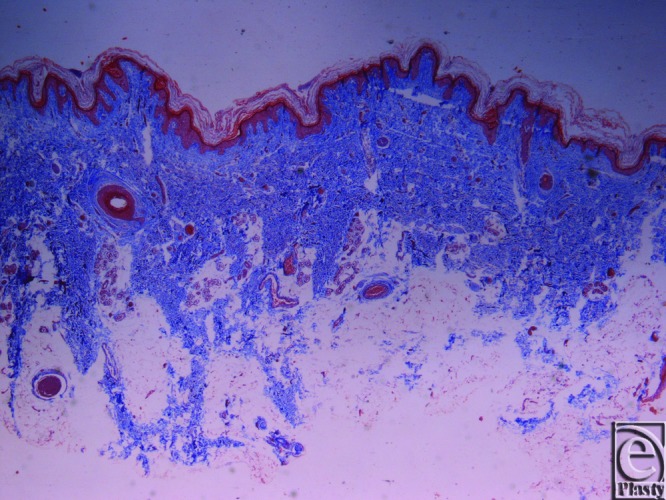
Inguinal crease. Masson trichrome stain ×25. The fibrocollagen bundles are thinner and less dense than in the inframammary and infragluteal creases.

**Figure 11:**
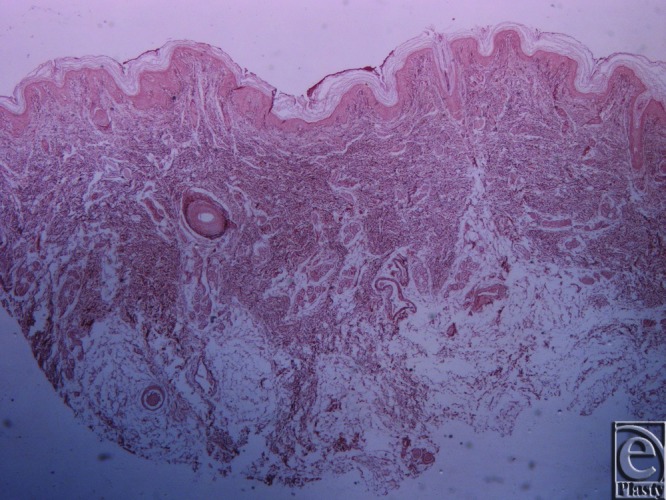
Inguinal crease. Orsein stain ×25. The elastic fibers are long and linear at the fibroelastic bundles and have a parallel distribution to the dermis.

**Figure 12:**
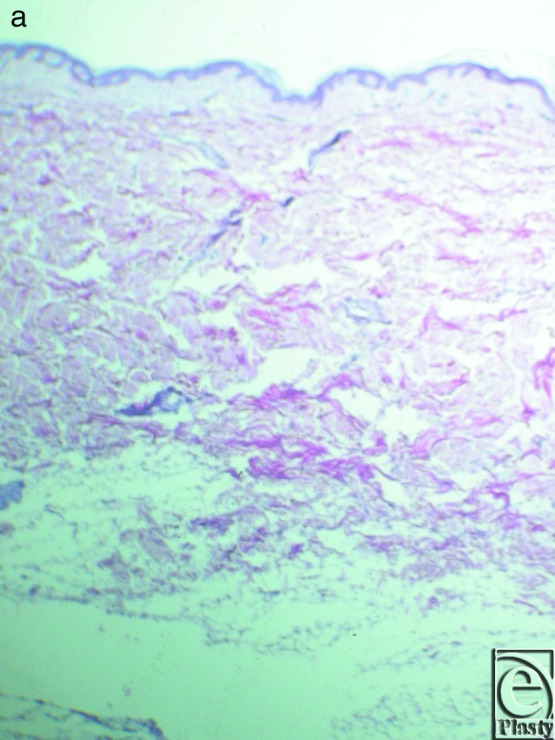

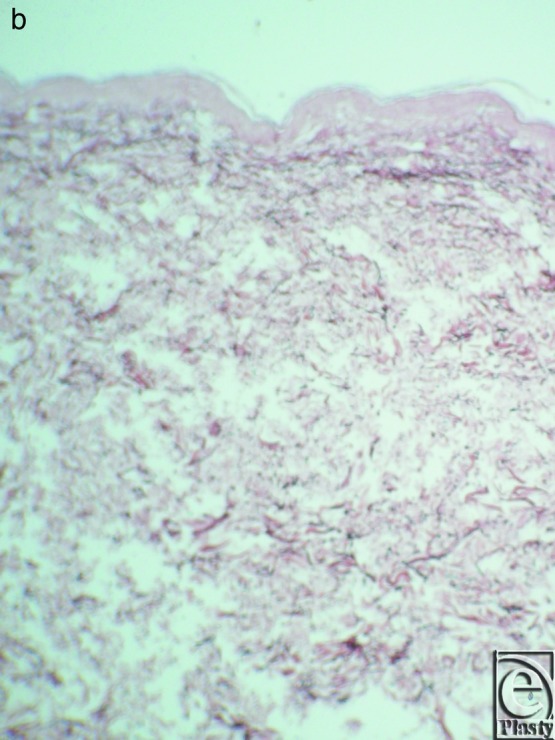
(a) Skin superior to the inframammary crease. Hematoxylin and eosin stain ×25. There is no beehive pattern of the collagen bundles with the subcutaneous fat tissue. (b). Skin inferior to the inframammary crease. Orsein stain ×100. The elastic fibers that are stained in black distribute in a random pattern.

Skin creases of the trunk^1^ (Fig 1) include the inframammary crease, the suprapubic crease, the oblique skin crease of the groin, and the infragluteal crease. The anterior axillary fold is also included in this project in comparison with the inframammary, infragluteal, and inguinal creases.

The inframammary crease^5^^,^^13^ lies over the fifth to sixth ribs. It is the lower boundary of the breast and is an important element of the natural ptotic appearance of the breast. This crease is among the few extensively studied creases, and it is more frequently found in literature as “inframammary fold”.^3^^,^^11^ There are controversies in the literature regarding the existence of a ligamentous band in the area of the crease. Bayati et al^14^ identified the “inframammary crease ligament” that originates from the fifth rib periosteum medially and the fascia between the fifth and sixth ribs laterally and extends into the dermis in the area of the inframammary crease.

Cooper^15^ has described the division of the superficial thoracic fascia into the superficial and deep layer of the fascia mammae, the breast being encapsulated among the 2 layers.

Van Straalen et al^6^ support the existence of a true inframammary ligament, whereas Maillard and Garey^16^ refer to a crescent-like ligamentous band, the “prepectoral ligament” that extends from the pectoralis major fascia to the skin of the inframammary crease.

The description of the SFS by Lockwood,^4^ its anatomy, and its relationships to the skin, fat, and musculoskeletal system may explain the formation of the creases, plateaus, valleys, and bulges of the body. The SFS is a network of connective tissue extending from the subdermal to the underlying muscle fascia. The connective tissue sheets are separated by layers of fat and are interconnected by vertical and oblique fibrous tissue septae. In areas of the body where the SFS adheres to the musculoskeletal surface, the zones of adherence are formed. In the breast, the SFS splits and forms the anterior and posterior lamellae of the breast, the former extending to the pectoral fascia and the latter to the dermis, fixing the breast to the overlying skin.

Garnier et al^17^ have studied the inframammary crease with detailed anatomical, radiological, ultrasound, and histological methods. Radiologically, they confirmed that the inframammary crease lies at the level of the sixth rib. Histologically, they referred to fibers extending from the prepectoral fascia to the superficial fascia, without any connections between the superficial fascia and the dermis at the level of the crease; thus, no ligamentous ligament was identified in the region.

Muntan et al^18^ described the connection of the dermis in the crease area with the superficial fascia in a variety of configurations. In some cases, the collagen fibers that arise from the superficial fascia level insert into the dermis or reach the level inferior to the dermis, and, in some cases, the deep fascia creates a fusion of the superficial fascia and the dermis at the level of the crease. The collagen bundles are distinct from Cooper's suspensory ligaments that can be found more superiorly in the breast gland.

Boutros et al^3^ support that the inframammary ligament theories have their “shortcomings” and prove that there is a “specific and unique” dermal structure that is responsible for the inframammary crease and that this structure is supported by the SFS. They demonstrate dense collagen bundles, dominant at all levels of the dermis, more pronounced in the papillary dermis, and running parallel to the long axis of the inframammary crease. The support of the SFS derives from the formation of a zone of adherence between the dermis and the underlying pectoralis fascia, holding this dermal structure in place.

The histological results of the specimens of the inframammary crease presented in this study are similar to the results of the previously described studies by Boutros et al^3^ and Lockwood.^4^ In the subcutaneous tissue, there is a well-defined network of dense collagen fibers that creates a beehive pattern network of the subcutaneous fat. This network is denser in deeper sites related to the pectoralis muscle fascia. The collagen fibers network has a broad base of attachments to the dermis. In the medial sites of the crease, the beehive pattern has the same structure as described earlier, only looser, which also becomes denser in the deeper sites. In the lateral sites of the crease, the beehive pattern network is just as well organized, with broad base attachments of the collagen bundles to the dermis. In some of the cases, the bundles are thinner than those at the other sites. The elastic fibers participate in the collagen pattern and radiate in a relatively parallel pattern in the reticular dermis and in a perpendicular fashion in the papillary dermis.

In addition, the role of the main supporting system of the breast, the suspensory ligament of Würinger, has to be acknowledged in the formation of the inframammary crease. This anatomical structure is U-shaped, attached to the thoracic cage, stretching from the fifth rib to the nipple as a horizontal septum and extending medially and laterally upward as vertical ligaments. Medially, it is attached to the sternum as high as the manubrium and laterally along the lateral border of the pectoralis minor muscle to the chest wall into the axilla, supporting the breast like a hammock. The most inferior border of this ligament and the overall configuration and “response” of the breast to its function contribute to the curvature, position, and shape of the inframammary fold. Addressing the reconstruction of the fold, consequently, includes the provision of a similar suspensory mechanism for the breast parenchyma.

The inguinal crease^1^^,^^5^ or Poubart's line,^10^ although believed to be the cutaneous reflection of the inguinal ligament, does not always directly correspond to the site of the inguinal ligament. The skin crease lies immediately superficial to the ligament in thin people, but in most of the population, it lies 2 to 3 cm distally.

In the specimens of the inguinal crease, the results of this study show a beehive pattern of fibrocollagenous network, thinner and looser in structure than the network of the inframammary and infragluteal creases. The elastic fibers are long and run along the collagen bundles and have a parallel course into the reticular dermis.

The infragluteal crease^1^ appears in literature as several terms such as “gluteal crease,” “infragluteal fold,” “infragluteal sulcus,”^19^ “horizontal gluteal fold,” “gluteal fold,” “inferior buttock crease,” “lower gluteal crease,” and “inferior gluteal crease.” This crease does not reflect the lower margin of the gluteus major muscle, but there are fibrous connections of the muscle surface with the deep fascia.

In the study by Babuccu,^19^ the results refer to strong fibrous bands that extend from the dermis of the medial one third of the fold to the ramus of the ischium and sacrum. They support that the anatomical structure is a fold in its medial part and a crease laterally. In the subcutaneous tissue, the fat and connective tissues of the SFS form a beehive pattern. This pattern extends to the lower limb under the crease along the lateral two-thirds. At the medial sites of the crease, the loose connective tissue of the septa forms dense collagen bundles. This pattern extends from the SFS to the deeper structures perpendicular to the dermis. In the central sites of the crease, there are insertions of striated skeleton muscle and its fascia to the dermal structures apart from the collagen bundles.

The results of the present study of the fresh cadaver specimens recognize in the subcutaneous tissue a well-defined network of dense collagen fibers in a beehive pattern network. The fibrous collagen bundles are well organized, thick, and dense, with a significant number of fibroblasts, particularly at the sites reflecting the center of the crease. In sections of the specimens from the medial and lateral sites of the crease, the collagen pattern is thinner, the presence of fibroblasts is not as significant as in the central sites of the crease, and the participation of the reticular fibers is less.

The elastic fibers are thick and participate in the fibrous bundles. They are denser in sections of the center of the crease. They radiate into the dermis in a parallel pattern (in the reticular dermis) and in a perpendicular fashion in the papillary dermis.

The anterior axillary fold^1^ was included in the study. This fold is formed along the lateral side of the pectoralis major muscle. The histological studies of this anatomic feature show a less organized structure of fibrocollagenous bundle pattern compared with the earlier described structure of the inframammary and infragluteal creases. The bundles are fragmented and not in a parallel pattern.

There is absence of any organized structure of the elastic fibers in the dermis along the anterior axillary fold. The absence of the structured beehive pattern of the fibroelastic tissue connecting the dermis to the deeper structure is a strong indication that this structure should be considered a fold and not a crease.

The suprapubic crease,^1^ located above the mons pubis, which is a strong zone of adherence, and the oblique skin crease of the groin,^1^ which is the flexure line of the hip joint and passing over the top of the great trochanter, were not included in this histological comparison study.

This study has included the histological comparison of the skin superior and inferior to the crease sites with the crease specimens. The collagen and elastic fibers have a rather random pattern, whereas at the crease site, there is a particularly organized network of fibroelastic bundles.

In specimens of the skin superior and inferior to the infragluteal crease, the elastic fibers are fewer and orientated parallel to the dermis.

The difficulty achieving methods of a satisfying reconstruction of the creases that are interrupted either by surgery or by other trauma is relevant to the specific histological characteristics of their formation. The skin creases are formations in that their maintenance depends on a system of attachments to the underlying fascia, as we refer to the creases of the trunk, with the participation of collagen and elastic fibers.

Wechselberger et al^20^ proposed a reconstruction method for the inframammary crease through elevation and advancement of a deepithelialized skin flap, attached to the periosteum of the fifth and sixth ribs. Pinnella^21^ proposed liposuction at the site of the inframammary crease to create a folding over the lower pole of the breast for reconstruction of the crease, whereas Ryan^22^^,^^23^ proposed advancement of a lower thoracic flap for postmastectomy reconstruction. Massiha^24^ introduced a capsular flap following deformities after aesthetic and reconstructive breast surgery.

Most of the methods proposed for reconstruction of the inframammary crease depend on the mechanism of scar tissue formation between the dermis and the underlying fascia or suspension of the underlying fascia to the periosteum. However, the lack of the elastic component in the reconstructed crease attachments may explain the unsatisfactory results of most of the aforementioned reconstructive methods. The knowledge of the histological structure of the creases of the body is important in the reconstruction of the relevant area.^2^^,^^3^ This study of histology of the skin creases of the trunk with the emphasis on the elastic fibers component can contribute method designs to be proposed for reconstruction of the relevant creases.

## CONCLUSIONS

Creases of the trunk are formed by well-organized collagen bundles in a beehive pattern, attached to the dermis and related to the underlying muscle fascia. The anterior axillary fold has less organized collagen bundles and elastic fiber participation and orientation. The elastic fibers participate in the formation of the collagen network and radiate in a parallel fashion in the reticular dermis and in a perpendicular pattern in the papillary dermis. The skin superior and inferior to the creases lacks such organization of the collagen and elastic fibers and is deposited in a random pattern.

Reconstruction of these features of the skin has an integral role in the function and form of the body and requires sufficient understanding of their structural (skin) anatomy as well as their anatomical and functional interactions with adjacent ligamentous structures and soft tissues.
